# Adaptation of Plants to UV-B Radiation with Altitude in Tuha Basin: Synergistic Regulation of Epidermal Structure, Secondary Metabolites, and Organic Element Allocation

**DOI:** 10.3390/life15091375

**Published:** 2025-08-29

**Authors:** Xiao-Min Wang, Guo-Qiang Zhao, Jie Chen, Fang-Zheng Jing, Li Li, Yu-Ying Wang, Ping Ma, Yu-Hang Wu, Shi-Jian Xu, Wen-Liang He

**Affiliations:** 1 Ministry of Education Key Laboratory of Cell Activities and Stress Adaptations, Gansu Province Key Laboratory of Gene Editing for Breeding, School of Life Sciences, Lanzhou University, Lanzhou 730000, China; wangxiaomin@lzu.edu.cn (X.-M.W.); zhguoqiang2024@lzu.edu.cn (G.-Q.Z.); chenjie2021@lzu.edu.cn (J.C.); jingfzh2024@lzu.edu.cn (F.-Z.J.); 13919265560@163.com (L.L.); 19811766627@163.com (Y.-Y.W.); 220220933580@lzu.edu.cn (P.M.); wuyh2023@lzu.edu.cn (Y.-H.W.); 2 State Key Laboratory of Plateau Ecology and Agriculture, Qinghai University, Xining 810016, China

**Keywords:** altitude, cuticle, epidermal hair, stomata, Turpan-Hami Basin, UV-B absorbing substances

## Abstract

Ultraviolet B (UV-B) radiation is a key environmental factor that limits plant growth and development. High UV-B intensity is a typical environmental feature in Turpan-Hami (Tuha) Basin in Xinjiang, China. In this study, the altitude-dependent UV-B adaptation strategies of plants in Tuha Basin were analyzed. Chlorophyll (Chl) and flavonoid (Fla) play an important role in absorbing UV-B radiation, scavenging free radicals, and maintaining photosynthetic performance under UV-B stress. Principal component analysis indicated that the total chlorophyll (Chl t), Chl a, Chl b, and Fla contents and the Chl a/Chl b ratio are important indicators for evaluating plant tolerance to UV-B. Noticeably, with increased altitudes, the roles of Chl b, Chl a/Chl b, and Fla become markedly significant. The characteristics of stomata, epidermal hair, and wax layer are closely correlated with the UV-B amount that reaches leaves. Epidermal hair density and cuticle thickness in leaves decreased with increased altitudes, whereas hydrogen oxide (H_2_O_2_) was significantly accumulated, but superoxide anion (O_2_^−^) remained unchanged. High altitude significantly increased the stomatal apparatus area, density and specific leaf area. Moreover, plants without epidermal hair had a larger stomatal apparatus area compared with plants with epidermal hair. However, the presence or absence of epidermal hair had no effect on cuticle thickness, H_2_O_2_ and O_2_^−^ levels. The carbon (C), nitrogen (N), and hydrogen (H) contents were high in plant leaves at high altitude, but the sulfur (S) content and C/N ratio were low. Taken together, plants in Tuha Basin could cope with UV-B radiation by synergistically regulating epidermal structures and synthesis of secondary metabolites. Meanwhile, these plants could further allocate and reconstruct organic elements to optimize their resource distribution in adaptation to UV-B radiation with different altitudes.

## 1. Introduction

Tuha Basin, a collective term for the Turpan Basin and the Hami Basin, is located in the eastern part of Xinjiang, China, bordering the Junggar Basin in the southwest and the Tarim Basin in the northwest. It belongs to a typical arid and semi-arid continental climate. The lowest point of the Tuha Basin (−154 m) is one of the hottest and driest places on Earth; however, the Bogda Peak in the north has an elevation of 5445 m [1]. The drastic terrain changes and extreme climate conditions (low annual precipitation, high evaporation, strong solar radiation) have nurtured diverse ecosystems from arid deserts to alpine glacier zones [2]. The eastern part of Xinjiang has a relatively long annual sunshine duration (2900–3450 h; [3]). Moreover, the Hami Basin has the highest solar radiation intensity, and its annual total solar radiation reaches 403.3 kJ cm^−2^ a^−1^, of which UV radiation reaches 28.231 kJ cm^−2^ a^−1^. Thus, the desert plants in the Tuha Basin have evolved unique adaptation mechanisms, forming structurally simple but diverse desert plant communities [4], which provide valuable resources for studying the adaptive evolution of plants under combined stresses of high altitude, UV radiation, drought stress and so on.

About 7% of solar radiation falls in the UV range (200–400 nm), and UV-B radiation (280–315 nm) is an inherent component of sunlight that reaches the earth’s surface [5]. UV-B radiation intensity increases with altitude, resulting in differential UV-B exposure for plants at different altitudes. Fu [6] reported that UV-B radiation on the northern slope of the Tianshan Mountains in Tuha basin increases with altitude, with an average increase of 1.185 MJ m^−2^ a^−1^ per 100 m elevation. Although UV-B serves as an important light signal involved in regulating photomorphogenesis, radiation stress adaptation, and floral transition [7], excessive exposure to UV-B can seriously affect plant development, leading to UV-B stress, i.e., reduced plant height and leaf area, shortened internodes, thickened leaves, delayed flowering, inhibition of pollen germination, and reduction in transpiration rate [8,9,10]. UV-B can also inhibit chlorophyll biosynthesis and promote its degradation [11], destroy the photosystem II (PSII) reaction center, reduce the maximum quantum yield (Fv/Fm) of PSII [12], leading to plant yellowing and slow growth [13]. High UV-B radiation causes cellular damage through multiple pathways, for example, reactive oxygen species (ROS) burst, lipid peroxidation, DNA and protein damage, ultimately leading to cell death [14]. For example, UV-B can induce DNA damage through the formation of pyrimidine dimer, finally impairing DNA synthesis and replication [15]. At the molecular level, UV-induced DNA damage can be efficiently repaired by photolyase. Among these, the CPD photolyase (*PHR*) expression is regulated by the UV-B-dependent UVR8 signaling pathway, blue light and UV-A radiation [16]. 

To alleviate the damage caused by UV-B radiation, plants have evolved a set of physiological and molecular adaptation mechanisms, mainly involving reducing exposure, protection, shielding, and repair. Plants can reduce leaf area and induce leaf curling to minimize exposure. Meanwhile, changes in wax composition in cuticles, accumulation of UV-B absorbing pigments, and leaf thickening collectively enhance the ability of plants to shield against UV-B radiation [9]. Plants can reduce PSII degradation caused by high light through adjusting chloroplast distribution and repair it through PSII recombination [17,18]. Flavonoids (Fla), i.e., flavanols and anthocyanins (Ant), can directly absorb UV-B in the epidermis [13]. Ant, as natural antioxidants, can protect plants from excessive UV-B radiation through scavenging free radicals [19,20]. Moreover, Ant can enhance the total antioxidant capacity by increasing L-ascorbic acid and glutathione levels [21,22]. After the removal of epidermal hairs, the photosynthetic electron transfer rate in *Verbascum thapsus* L. is significantly inhibited under UV-B radiation [23]. The cuticle of leaf epidermis is composed of a series of very-long-chain fatty acids (VLCFA) and their derivatives [24], which is crucial for preventing non-stomatal water loss and damage caused by UV-B radiation, insects, and fungi [25,26,27]. The UV rays that reach the surface of plants can be reflected or scattered by epidermis and cuticles and absorbed by phenolic compounds in cuticles [28]. Meanwhile, phenolic substances present in cuticles play a crucial role in photoprotection under UV-B radiation, especially in the UV-C and UV-B ranges. Zhou et al. (2024) reported that calcium signal plays a significant role in UV-B responses by modulating phenolic acid accumulation to enhance plant UV-B tolerance [29]. Recent studies reveal that the UVR8 protein serves as the primary UV-B receptor in plants. UVR8 activation initiates downstream signaling pathways [30]. Under UV-B exposure, UVR8 undergoes monomerization and interacts with COP1 to facilitate the accumulation of HY5, a key transcription factor that regulates the expressions of numerous UV-B -associated genes [31].

Stomata serve as main channels for gas exchange between leaves and environments, and play a major role in regulating water and C cycle [32]. Plants can optimize transpiration and photosynthetic efficiency by regulating stomatal aperture to adapt to different environmental stresses, such as drought, salt, high light, UV-B radiation, etc. [33,34]. The changes in stomatal size, distribution and density are also key adaptive characteristics of plants in response to environmental stresses [35,36,37]. Yang et al. (2014) reported that the stomatal density of species in the Qinghai–Tibet plateau is significantly higher than that in the Mongolian grasslands (mean difference is 23%), while the stomatal size is smaller, reflecting a trade-off between photosynthetic efficiency and moisture preservation in high-radiation environments [36]. With the increase in altitude as well as the decrease in temperature and partial pressures of CO_2_ and O_2_, stomatal density changes accordingly; moreover, the pattern exhibits strong plasticity [36,37,38,39]. The lower temperatures in high-altitude areas slow down metabolic processes, while the lower levels of CO_2_ challenge the ability of plants to effectively carry out photosynthesis. Therefore, plants may reduce stomatal density to minimize water loss caused by transpiration and optimize photosynthetic performance. However, the characteristics and mechanism of plant stomata changing with altitude and UV-B radiation is still unclear.

To elucidate the altitude-dependent UV-B adaptation strategies of plants, the UV-adaptation mechanism of 78 plant species was systematically analyzed through detecting the UV-B tolerance- related physiological and morphological indicators under different altitudes in the Tuha Basin. On this basis, the longitudinal adaptation strategies and underlying mechanisms of plants adaptation to changes in altitude and UV-B radiation were investigated. Our results suggest that plants could cope with UV-B radiation by synergistically regulating epidermal structures (epidermal hairs, cuticle, and stomatal characteristics), synthesis of secondary metabolites (chloroplast pigments and Fla), and allocation and reconstruction of organic elements (C, N, S) under different altitudes in the Tuha Basin.

## 2. Materials and Methods

### 2.1. Study Area and Sample Collection

The Turpan-Hami Basin (the Tuha Basin) is located in Xinjiang, China (91°06′33″–96°23′00″ E, 40°52′47″–45°05′33″ N). Tuha Basin is approximately 660 km from east to west and 60–100 km from north to south, with a total area of about 53,500 km^2^. The Tuha Basin region belongs to a typical continental arid climate characterized by extreme drought and intense UV-B radiation, which forms unique and diverse resources of anti-adversity plants. To investigate the mechanisms of plant adaptation to UV-B radiation at different altitudes in the Tuha Basin, representative plant samples were collected in June 2023 and June 2024 at different altitudes (400–2500 m) in the East Tianshan Mountains in the Tuha Basin, and categorized into three groups, i.e., low-altitude region (LA; <1100 m), middle-altitude region (MA; 1100–1900 m) and high-altitude region (HA; >1900 m) based on the distribution characteristics of vegetation (Tables S1 and S2). In each sampling area, all plant species were collected with at least 6 replicates.

### 2.2. Chlorophyll Content Analysis

The chlorophyll content was detected according to Sartory and Grobbelaar [40]. Briefly, 25 mg of leaves was cut into pieces and extracted with 1 mL of 95% ethanol at 4 °C for 72 h in the dark. The absorbance at 470, 663 and 645 nm was determined, the contents of chlorophyll a (Chl a), chlorophyll b (Chl b), total chlorophyll (Ch t) and carotenoid (Car) were calculated according to the followed formula.

Chl a (μg/mL) = 12.7 OD_663_ − 2.69 OD_645_

Chl b (μg/mL) = 22.9 OD_645_ − 4.68 OD_663_

Chl t (μg/mL) =8.02 OD_663_ + 20.2 OD_645_

Car (μg/mL) = (1000 OD_470_ − 1.82 Chl a − 85.02 Chl b)/198

### 2.3. Analysis of Anthocyanin Content

The anthocyanin (Ant) content was detected according to Vandenbussche et al. [41]. 25 mg of leaves were extracted with 600 μL of acidic methanol (methanol/HCl = 99:1, *v*/*v*) at 4 °C for 72 h. After adding 400 μL of chloroform and 400 μL of water into the supernatant, the mixture was centrifuged at 10,000× *g* for 10 min at 4 °C. The absorbance of the supernatant was measured at 530 nm. Ant content was expressed as OD530/gFW.

### 2.4. Flavonoid Content Analysis

The flavonoid (Fla) content was determined with the Plant Flavonoids kit (Keming Biotechnology, Suzhou, China). After the leaves were dried, 0.2 g of dried samples were extracted with 2 mL of 60% ethanol at 60 °C for 2 h with shaking. After centrifuged at 10,000× *g* for 10 min, the supernatant was collected for analysis. The Fla content was determined according to the instructions of the kit. The Fla content (mg/gDW) was calculated based on a standard curve (y = 5.02x + 0.0007, R^2^ = 0.9996)

### 2.5. Analysis of Cuticle Thickness

The cuticle was analyzed according to Tanaka et al. [42]. Plant leaves were immersed in 0.5% of toluidine blue (TB) solution, vacuum-infiltrated for 30 min, and then incubated in darkness for 10 h. The cuticle thickness was quantified using Image J 6.0.

### 2.6. Statistics of Epidermal Hair Density

The morphology of epidermal hair of leaves was observed and photographed with the stereo microscope [43]. The density of epidermal hair (/mm^2^) was counted by using the TCapture software.

### 2.7. Analysis of Stomatal Features

The stomatal features were analyzed according to Han et al. [44]. The leaf segments (0.5 cm × 0.5 cm) from different parts (tip, margin, base) were fixed in fixative solution (ethanol/acetic acid = 3:1, *v*/*v*) (≥1 h), rinsed, and photographed under a light microscope. Stomatal apertures (length, μm; width μm; area, μm^2^; density, /mm^2^) on the abaxial surface were measured using Image J 6.0, and data was averaged as the final result.

### 2.8. Analysis of H_2_O_2_ and O_2_^−^ Contents

The H_2_O_2_ content was analyzed according to Wang et al. [45]. The leaves were incubated in 3,3′-Diaminobenzidine (DAB) buffer containing 1 mg/mL DAB, 50 mM Tris-acetic acid, pH 5.0, and 0.05% TritonX-100 in the darkness at room temperature for 4 h. After chlorophyll was completely removed from the stained leaves with 75% ethanol at 75 °C, the samples were photographed. The H_2_O_2_ content was quantified using Image J 6.0.

The O_2_^−^ content was analyzed according to Jha et al. [46]. The leaves were incubated in nitroblue tetrazolium (NBT) buffer containing 10.5 mg/mL NBT, 50 mM PBS, pH 7.4, and 0.05% TritonX-100 in the darkness at room temperature for 3 h. After chlorophyll was removed from the stained leaves with 75% ethanol at 75 °C, the samples were photographed. The O_2_^−^ content was quantified using Image J 6.0.

### 2.9. Determination of Organic ELEMENT Content

Determination of organic element content in leaves was carried out following the method of Zhao et al. [47]. Leaves were initially dried at 105 °C for 30 min, then transferred to a 65 °C oven for 48 h and dried to constant weight. After the dried samples were uniformly ground using a ball mill, element content (%) was determined using an elemental analyzer (Elementar Vario EL cub, Elementar, Langenselbold, Germany).

### 2.10. Comprehensive Evaluation Based on Membership Function

The comprehensive evaluation method based on membership function was used to evaluate the UV-B tolerance in the selected species [48]. Firstly, fuzzy membership function value for each species was calculated based on Formula (1); secondly, the PCA was performed using IBM SPSS Statistics 21 to determine the correlation coefficient (r) and contribution rate between each principal component and each indicator; thirdly, a principal component membership function value was calculated based on Formula (2); finally, total membership function value (D value) was calculated based on Formula (3). After the sensitivity analysis, D value is used to evaluate the UV-B tolerance of species.

W_ij_ = (X_ij_ − X_min_)/(X_max_ − X_min_) (1)

X_max_: the maximum value of an index; X_min_: the minimum value of an index; X_ij_: the absolute value of an index.

Uj = ∑^n^_j_ = _1_W_ij_R_ij_ (2)

R_ij_: the r between index i and index j.

D = ∑^n^_j_ = _1_U_j_x P_j_/(∑^n^_j_ = _1_P_j_) (3)

P_j_: the contribution rate of a principal component; ∑^n^_j_ = _1_P_j_: the sum of the contribution rates of the selected principal components.

### 2.11. Statistical Analysis

The data were statistically analyzed using the IBM SPSS 22.0 software and expressed as means ± SD (n ≥ 3). For the comparison of two groups, the significant analysis was performed with Student’s *t* test. For multiple comparisons, the significant analysis was performed with one-way ANOVA and Tukey’s test. The images were created by Origin 2024. PCA was analyzed with OriginPro 2021.

## 3. Results

### 3.1. Changes in UV-B Absorbing Substances and Chloroplast Pigments in Plants Across Altitudes in the Tuha Basin

To elucidate altitude-dependent UV-B adaptation strategies in plants, the plants collected from the Tuha Basin were classified into three groups: low-altitude (LA, <1100 m) plants, middle-altitude (MA, 1100–1900 m) plants, and high-altitude plants (HA, >1900 m; Tables S1 and S2). A total of 24 plant species were collected from the LA regions, including *Haloxylon ammodendron*, *Lycium ruthenicum*, and *Karelinia caspia* (Tables S1 and S2). Twenty-two species were collected in the MA regions, including *Phragmites australis*, *Pennisetum alopecuroides*, and *Carex ovatispioulata Thunb* (Tables S1 and S2). Thirty-two plant species were collected from HA regions, including *Oxytropis glabra*, *Potentilla chinensis*, and *Neotrinia splendens* (Tables S1 and S2).

**Figure 1:**
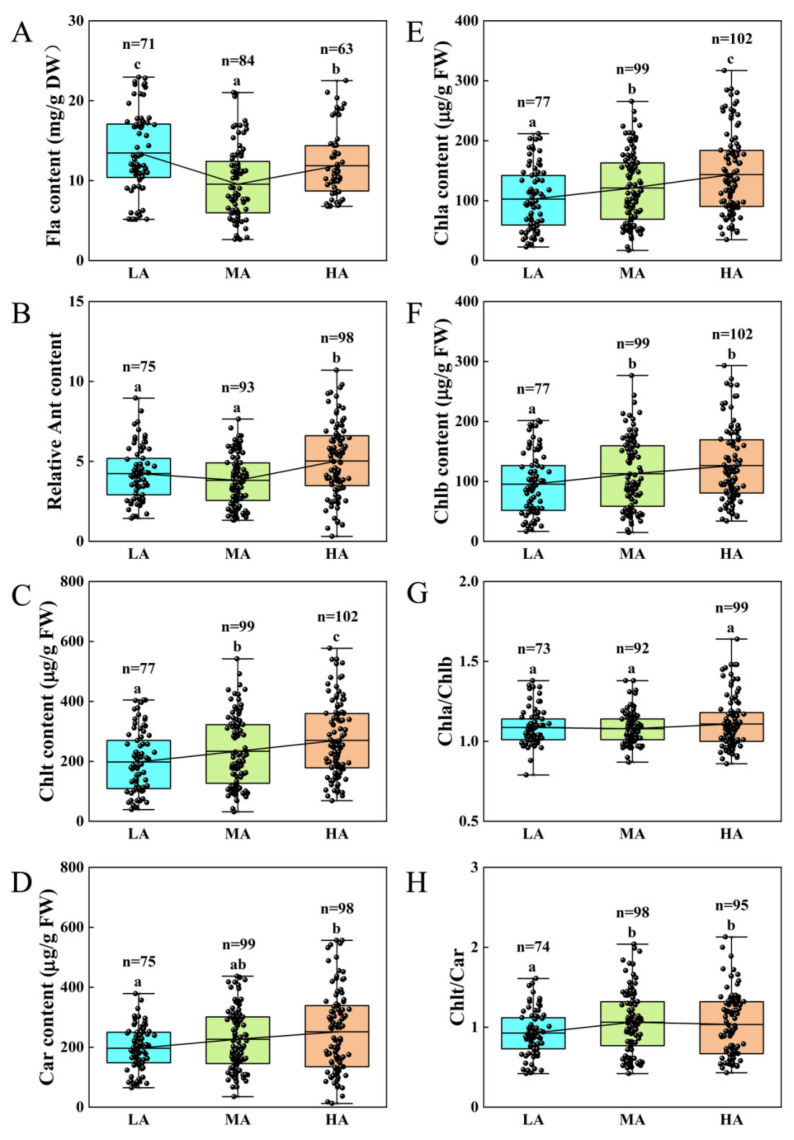
Analysis of chlorophyll pigment and flavonoid (Fla) contents in leaves of plants at different altitudes in the Tuha Basin. (**A**) Fla; (**B**) Ant; (**C**) Chl t; (**D**) Car; (**E**) Chl a; (**F**) Chl b; (**G**) Chl a/Chl b; (**H**) Chl t/Car. Different letters on the error bar indicate significant difference at *p* < 0.05. LA, low altitude; MA, middle altitude; HA, high altitude.

The Fla content was highest in the LA plants, followed by the HA plants, and lowest in the MA plants (Figure 1A; Table S3). Compared to the LA plants, the Fla content decreased by 11.88% in the HA plants and by 29.03% in the MA plants. The anthocyanin (Ant) content was highest in the HA plants, and it significantly increased by 18.63% and 32.02% compared to the LA and MA plants, respectively (Figure 1B; Table S3). The contents of chlorophyll a (Chl a), chlorophyll b (Chl b), total chlorophyll (Chl t), and Car increased with elevation. Compared to the LA group, the contents of Chl a, Chl b, Chl t and Car in the MA group were significantly increased by 18.03%, 18.19%, 18.11%, and 15.13%, respectively. Compared to the MA group, the HA group exhibited increases of 18.50% in Chl a, 12.05% in Chl b, 15.39% in Chl t, and 10.90% in Car (Figure 1C–F; Table S3). However, the Chl a/Chl b ratio showed no significant differences among the three groups (Figure 1G; Table S3). Comparatively, the Chl t/Car ratio increased significantly by 13.98% in the MA group and 10.75% in the HA group compared to the LA group; but there was no significant difference between the MA and HA groups (Figure 1D; Table S3). These results suggested that except for Fla, the contents of Ant, chlorophyll (Chl a, Chl b, Chl t), and Car showed consistent and significant increases with elevation, indicating that Ant and chloroplast pigments play important roles in plant adaptation to the changed UV-B environments with elevation in the Tuha Basin.

### 3.2. Principal Component Analysis (PCA) on the Physiological Indicators in Plant Tolerance to UV-B Under Different Altitudes

To further identify the key indicators in plant adaptation to the changing UV-B environments with increased altitudes in the Tuha Basin, and comprehensively assess their UV-B tolerance, PCA was performed on the eight physiological indicators, i.e., UV-B absorbing compounds (Fla, Ant), chloroplast pigments (Car, Chl t, Chl a, Chl b), Chl t/Car, and Chl a/Chl b. In the LA region, the first three axes of PCA explained 54.807%, 19.732%, and 13.145% of trait variation, respectively, with a cumulative contribution rate of 87.685%. The first principal component (λ = 4.358) was mainly represented by Chl a, Chl b, and Chl t, r = 0.977, 0.991, 0.987, respectively. It indicated that this principal component primarily reflects the variation in chlorophyll content in various plant species in the LA area of the Tuha Basin. The second principal component (λ = 1.579) was mainly represented by Chl a/Chl b (r = 0.714), indicating that it primarily reflects Chl a/Chl b variation. The third principal component (λ = 1.052) was mainly represented by Fla content (r = 0.695), indicating that it primarily reflects the variation in Fla content in various plant species in the LA area of the Tuha Basin (Table 1; Figure S1A).

In the MA regions, the first three axes of PCA explained 53.314%, 25.421%, and 10.430% of trait variation, respectively, with a cumulative contribution rate of 89.165%. The first principal component (λ = 4.265) was mainly represented by Chl a, Chl b, and Chl t, r = 0.975, 0.985, 0.985, respectively. It indicated that this principal component primarily reflects the variation in chlorophyll content. The second principal component (λ = 2.034) was mainly represented by Chl a/Chl b (r = 0.828), indicating that it primarily reflects the Chl a/Chl b variation. The third principal component (λ = 0.824) was mainly represented by Fla content (r = 0.744), indicating that it primarily reflects the variation in Fla content in the plant species in the MA area of the Tuha Basin (Table 1; Figure S1).

In the HA regions, the first three axes of PCA explained 45.635%, 30.247%, and 11.321% of trait variation, respectively, with a cumulative contribution rate of 87.203%. The first principal component (λ = 3.651) was mainly represented by chlorophyll content (Chl a, Chl b, Chl t), r = 0.877, 0.905, 0.946, respectively. It indicated that this principal component primarily reflects the variation in chlorophyll content. The second principal component (λ = 2.420) was mainly represented by Chl a/Chl b (r = 0.929), indicating that this component primarily reflects the Chl a/Chl b variation. The third principal component (λ = 0.906) was mainly represented by Fla content (r = 0.806), indicating that this component primarily reflects the variation in Fla content in the plant species in the HA area of the Tuha Basin (Table 1; Figure S1).

Based on PCA results, the chlorophyll content (Chl a, Chl b, Chl t), Chl a/Chl b ratio, and Fla content in low-, middle-, and high-altitude plants of the Tuha Basin cover most of the information on eight physiological indicators and are important indicators for evaluating UV-B tolerance of the plant species in the Tuha Basin. Noticeably, with the increased altitude, Chl b, Chl a/Chl b, and Fla played increasingly prominent roles (Table 1).

### 3.3. Comprehensive Evaluation of UV-B Tolerance in Tuha Plants Under Different Altitudes Based on Membership Function Analysis

To comprehensively evaluate the UV-B tolerance of typical plants in the Tuha Basin, the fuzzy mathematical membership function comprehensive evaluation method was used in combination with PCA results (Table 2). Based on the D values, Tuha plants at each altitude were classified into four categories: Class I (high UV-tolerant plants; D > 0.910), Class II (strong UV-tolerant plants; 0.500 < D < 0.910), Class III (moderate UV-tolerant plants; 0.300 < D < 0.500), and Class IV (low UV-tolerant plants; D < 0.300). Specifically, there were ten UV-tolerant plants (I and II) in the HA regions, including *Oxytropis glabra* (0.947), *Potentilla chinensis* (0.933), *Neotrinia splendens* (0.870), and *Medicago sativa* (0.859). There were 11 UV-tolerant plants (I and II) in the MA areas, including *Phragmites australis* (0.973), *Pennisetum alopecuroides* (0.959), *Carex ovatispioulata* (0.951), *Oxytropis glabra* (0.782), and *Caragana sinica* (0.771). There were six UV-tolerant plants (I and II) in the LA area, including *Sophora alopecuroides* (0.913), *Phragmites australis* (0.839), *Populus euphratica* (0.645), and *Apocynum venetum* (0.622). The low UV-tolerant plants included *Neotorularia humilis* (0.293) from the HA regions, *Neotrinia splendens* (0.279), *Anabasis brevifolia* (0.253), *Lycium ruthenicum* (0.224), and *Anabasis aphylla* (0.152) from the MA regions, and *Haloxylon ammodendron* (0.243), *Lycium ruthenicum* (0.283), and *Karelinia caspia* (0.217) from the LA regions (Table 2). It is noteworthy that *Braya humilis* in the HA region belongs to the low UV-tolerant plant; comparatively, it belongs to moderate UV-tolerant plant in the MA region (Table 2). These results indicate that the differences in stress tolerance of the same plant species in different environments might reflect an adaptive strategy to adapt to changing environments.

To clarify the biological significance of the total membership function values, linear regression analysis was performed between the total membership function values and the fuzzy mathematics membership functions of eight physiological indicators. As shown in Figure S2, in the LA regions, except for Chl a/Chl b, all other indicators showed significantly positive correlations with the total membership function. In the MA area, except for Fla and Chl a/Chl b, all other indicators exhibited highly and significantly positive correlations with the total membership function. In the HA area, except for Fla and Chl t/Car, all other indicators showed significantly positive correlations with the total membership function. These results demonstrate that the total membership function value can serve as a comprehensive indicator for evaluating UV tolerance of plant species in different altitudes of the Tuha Basin. Plants with high D values have stronger tolerance to UV environments.

### 3.4. Variations in Epidermal Hairs and Cuticles in Various Plant Species from Different Altitudes of the Tuha Basin

**Figure 2:**
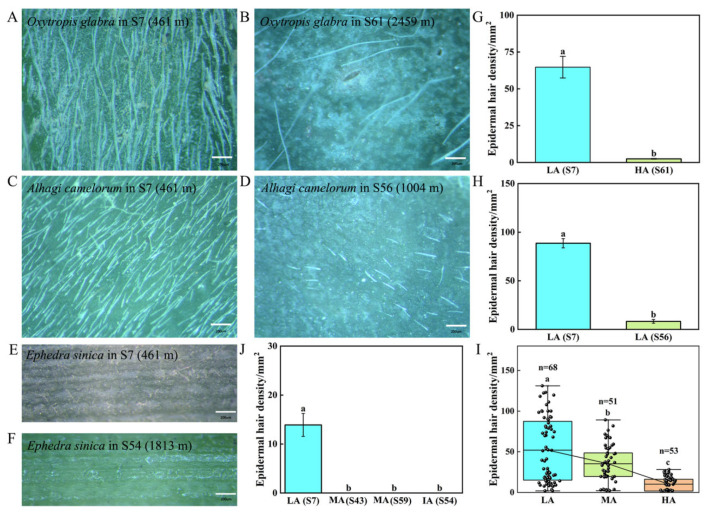
Analysis of epidermal hair density in plants at different altitudes in the Tuha Basin. (**A**–**F**) The morphology of epidermal hair of *Oxytropis glabra* from S7 (**A**) and S61 (**B**); *Alhagi camelorum* from S7 (**C**) and S56 (**D**); *Ephedra sinica* from S7 (**E**) and S54 (**F**). Bar = 200 μm. Quantification of epidermal hair density of *Oxytropis glabra* (**G**) in A and B, *Alhagi camelorum* (**H**) in C and D, *Ephedra sinica* (**J**) in E and F. (**I**) The variation trend of epidermal hair density at different altitudes. Different letters on the error bar indicate significant difference at *p* < 0.05. LA, low altitude; MA, middle altitude; HA, high altitude.

Research has demonstrated that epidermal hairs and cuticles play crucial roles in plant protection against UV-B radiation [23,49]. To investigate the variation in epidermal hairs with changing altitudes and UV-B intensity among Tuha plants, the epidermal hair density was further analyzed. For this purpose, the samples collected in 2024 were used for analysis, and based on the density of epidermal hairs, Tuha plants at each altitude were classified into four categories (Table S4): Class I (plants with high density of epidermal hairs; density > 60/mm^2^), Class II (plants with moderate density of epidermal hairs; 20/mm^2^ < density < 60/mm^2^), Class III (plants with low density of epidermal hairs; 10/mm^2^ < density < 20/mm^2^), and Class IV (plants with sparse epidermal hairs; density < 10/mm^2^). The results further showed that the density of epidermal hairs in *Oxytropis glabra* at S7 site (461 m) was 26.2 times higher than that in the same species at the HA site-S61 (2459 m; Figure 2A,B,G). Similarly, the density of epidermal hairs in *Alhagi sparsifolia* at the LA site-S7 (461 m) was 10.7 times higher than that in the same species at the MA site-S56 (1004 m; Figure 2C,D,H). *Ephedra sinica* only displayed sparse epidermal hairs at LA site-S7; comparatively, its epidermal hairs were completely absent at the MA site-S54 (1813 m; Figure 2E–J). Thus, as the altitude increased, the density of epidermal hairs continued to decrease within species in the Tuha Basin. Moreover, this pattern extended among species, as shown in Figure 2I and Table S4.

**Figure 3:**
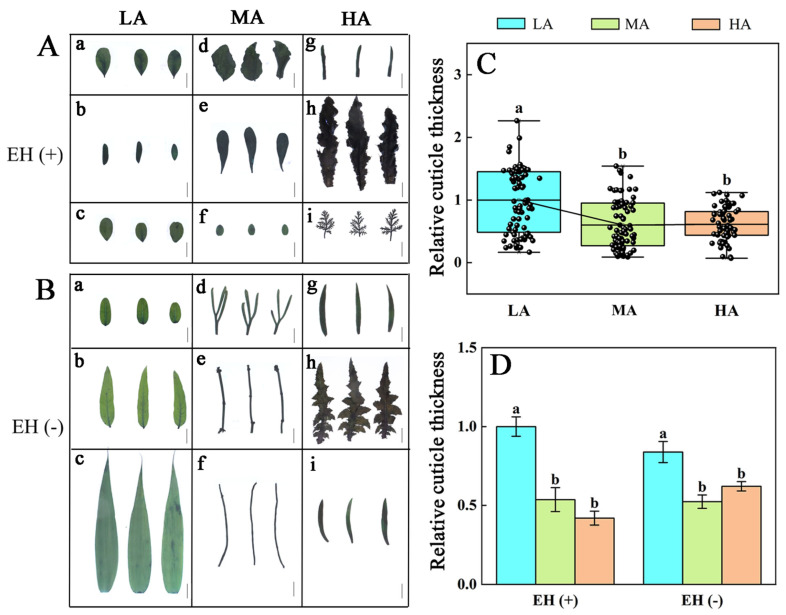
Analysis of cuticle thickness in plant leaves at different altitudes in the Tuha Basin. (**A**,**B**) TB-stained leaves of plants with (**A**, EH+) or without (**B**, EH−) epidermal hair at different altitudes. Bar = 1 cm. (**C**) Quantification of relative cuticle thickness at different altitudes. (**D**) Quantification of relative cuticle thickness in plants with (EH+) or without (EH−) epidermal hair at different altitudes. In **A**, **a**: *Alhagi camelorum* from S7 (461 m); **b**: *Oxytropis glabra* from S9 (651 m); **c**: *Alhagi camelorum* from S56 (1004 m); **d**: *Glycyrrhiza uralensis* from S58 (1139 m); **e**: *Nitraria sphaerocarpa* from S58 (1139 m); **f**: *Sphaerophysa salsula* from S58 (1139 m); **g**: *Silene gallica* from S60 (2093 m); **h**: *Cirsium arvense* from S61 (2459 m); **i**: *Artemisia* from S61 (2459 m). In **B**, **a**: *Karelinia caspia* from S9 (651 m); **b**: *Apocynum venetum* from S9 (651 m); **c**: *Phragmites australis* from S7 (461 m); **d**: *Peganum harmala* from S59 (1785 m); **e**: *Ephedra sinica* from S43 (1492 m); **f**: *Haloxylon ammodendron* from S7 (461 m); **g**: *Artemisia dracunculus* from S36 (2300 m); **h**: *Lactuca tatarica* from S61 (2459 m); **i**: *Galium spurium* from S61 (2459 m). Different letters on the error bar indicate significant difference at *p* < 0.05. LA, low altitude; MA, middle altitude; HA, high altitude.

The analysis of cuticle thickness revealed that in the Tuha Basin ecosystem, the cuticle thickness of leaves exhibited a decreasing trend with increased altitudes, although no significant difference was observed among plants from middle to high altitudes (Figure 3A–C; Table S5). To further investigate the influence of epidermal hairs on cuticle thickness, the samples were divided into two groups based on the presence or absence of epidermal hairs. The results demonstrated that the presence or absence of epidermal hairs had no significant effects on cuticle thickness at the same altitude (Figure 3D). Moreover, regardless of presence or absence of epidermal hairs, there was a significant decrease in cuticle thickness with increased altitude in the LA and MA areas. However, no significant difference was detected between the MA and HA areas (Figure 3D). These findings indicate that the variation in cuticle thickness was not significantly correlated with epidermal hair structure, and plant species in the Tuha Basin exhibited a decreasing trend in cuticle thickness with the increased altitude.

### 3.5. Stomatal Morphology in Various Plant Species from Different Altitudes of the Tuha Basin

**Figure 4:**
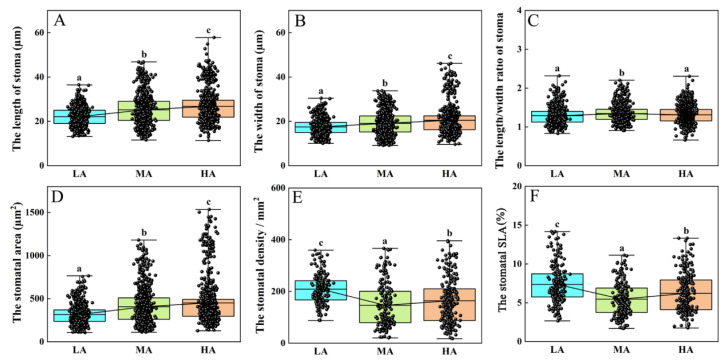
Analysis of stomatal characteristics in plant leaves at different altitudes in the Tuha Basin. (**A**) The length of stomata. (**B**) The width of stomata. (**C**) The length/width ratio of stomata. (**D**) The area of stomata. (**E**) The stomatal density. (**F**) The stomatal specific leaf area (SLA). Different letters on the error bar indicate significant difference at *p* < 0.05. LA, low altitude; MA, middle altitude; HA, high altitude.

Under UV-B radiation, UVR8, the UV-B receptor, interacts with COP1 to stabilize the HY5 protein, thereby participating in the regulation of stomatal development [50]. Based on this, we investigated the changes in stomatal morphology in Tuha plants from different altitude regions. The results revealed that as altitude increased, the length, width, and area of stomata were significantly increased (Figure 4A,B,D). Compared with the LA plants, the length, width, and area of stomata were increased by 14.08%, 9.17%, and 28.58% in the MA plants, respectively, and by 20.97%, 17.10%, and 43.95% in the HA plants, respectively (Figure 4A,B,D). This suggests that stomatal size and area were increased with elevated altitude and UV-B intensity. However, the length-to-width ratio of stomata did not follow the same trend, and it was significantly greater in the MA plants than that in the LA and HA plants (Figure 4C). The stomatal density exhibited an opposite trend (Figure 4E). Compared with the LA plants, the stomatal density was decreased by 42.54% in the MA plants and by 27.41% in the HA plants (Figure 4E). Similarly, the stomatal specific leaf area (SLA) in the MA plants was decreased by 34.94% compared with that in the LA plants and by 13.83% in the HA plants (Figure 4F).

**Figure 5:**
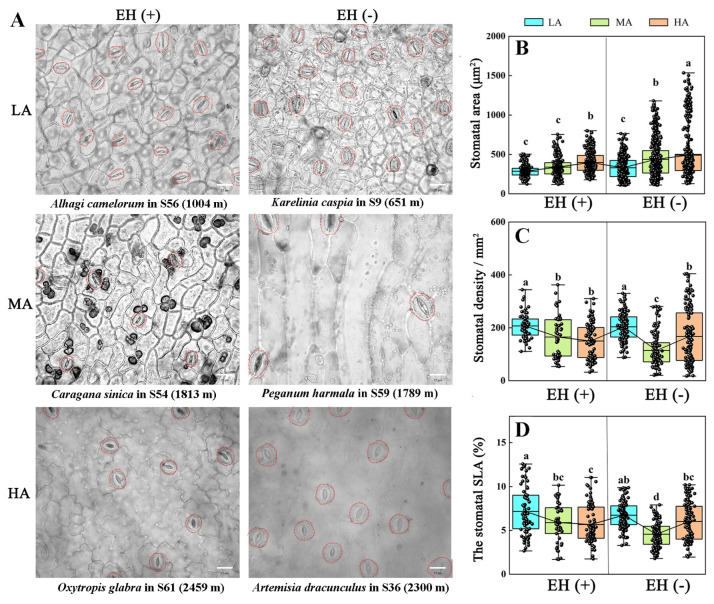
Effects of epidermal hairs on stomatal characteristics in plants at different altitudes in the Tuha Basin. (**A**) Phenotypes of stomata in plants with (EH+) or without (EH−) epidermal hairs at different altitudes. Bar = 25 µm. (**B**–**D**) Quantification of the stomatal area (**D**), stomatal density (**C**), and stomatal SLA (**D**) in plants with (EH+) or without (EH−) epidermal hairs at different altitudes. Different letters on the error bar indicate significant difference at *p* < 0.05. LA, low altitude; MA, middle altitude; HA, high altitude.

The influence of epidermal hairs on stomatal morphology of Tuha plants at different altitudes was further analyzed. The results showed that, in the LA regions, the presence or absence of epidermal hairs had no significant effect on stomatal area (Figure 5A,B), stomatal density (Figure 5A,C), or stomatal SLA (Figure 5A,D). However, in the MA regions, Tuha plants without epidermal hairs had significantly larger area of stomatal apparatus (26.39%), lower stomatal density (30.72%) and SLA (44.21%) compared with Tuha plants with epidermal hairs (Figure 5). In the HA regions, Tuha plants without epidermal hairs had significantly larger area of stomatal apparatus (19.67%) compared with Tuha plants with epidermal hairs (Figure 5A,B). However, there were no significant differences in stomatal density or SLA (Figure 5A,C,D). These results indicated that increase in altitude induces stomatal enlargement and enhances stomatal density and SLA. Additionally, in the middle- and high-altitude regions, Tuha plants without epidermal hairs tended to develop larger stomata compared to Tuha plants with epidermal hairs, but with a reduction in the stomatal density and SLA. The increased stomatal area, along with decreased density and SLA at higher altitudes, may contribute to reduced transpiration rate of plants.

### 3.6. Difference in ROS Contents in PLANT Species from Different Altitudes of the Tuha Basin

**Figure 6:**
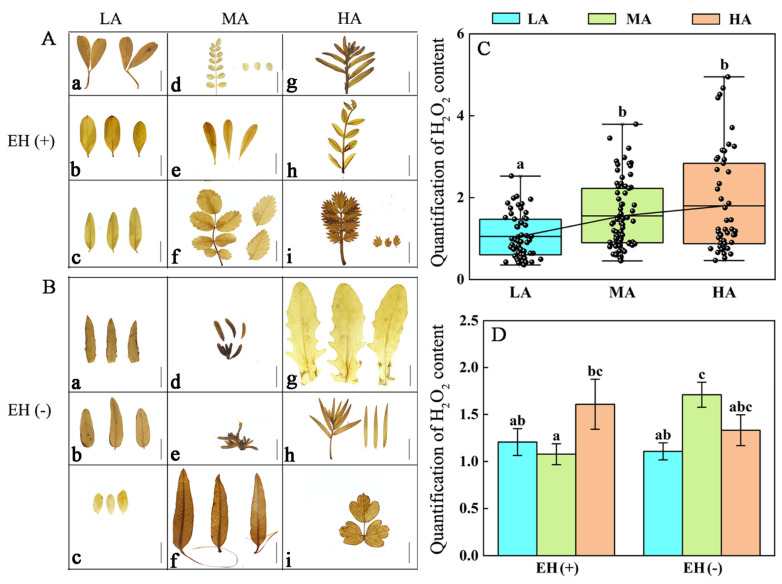
Analysis of hydrogen peroxide (H_2_O_2_) in plant leaves at different altitudes in the Tuha Basin. (**A**,**B**) DAB staining of H_2_O_2_ in leaves of plants with (**A**, EH+) or without (**B**, EH−) epidermal hairs at different altitudes. Bar = 1 cm. (**C**) Quantification of H_2_O_2_ in plant leaves at different altitudes. (**D**) Quantification of H_2_O_2_ in plants with (EH+) or without (EH−) epidermal hairs at different altitudes. In **A**, **a**: *Caragana halodendron* from S7 (461 m); **b**: *Alhagi camelorum* from S7 (461 m); **c**: *Sophora alopecuroides* from S9 (651 m); **d**: *Sphaerophysa salsula* from S58 (1139 m); **e**: *Nitraria sphaerocarpa* from S58 (1139 m); **f**: *Rosa laxa* from S58 (1139 m); **g**: *Silene gallica* from S60 (2093 m); **h**: *Hedysarum* from S60 (2093 m); **i**: *Argentina anserina* from S61 (2459 m). In **B**, **a**: *Rhaponticum repens* from S7 (461 m); **b**: *Karelinia caspia* from S9 (651 m); **c**: *Zygophyllum pterocarpum* from S7 (461 m); **d**: *Salsola collina* from S54 (1813 m); **e**: *Halogeton glomeratus* from S59 (1785 m); **f**: *Apocynum venetum* from S58 (1139 m); **g**: *Thlaspi arvense* from S60 (2093 m); **h**: *Artemisia dracunculus* from S60 (2093 m); **i**: *Thalictrum aquilegiifolium* from S60 (2093 m). Different letters on the error bar indicate significant difference at *p* < 0.05. LA, low altitude; MA, middle altitude; HA, high altitude.

UV-B radiation can induce ROS production, thereby activating the plant antioxidant system; meanwhile, changes in ROS levels can influence gene expressions and subsequently regulate metabolism and metabolite levels in plants to adapt to UV-B stress [51]. Therefore, we investigated changes in ROS (H_2_O_2_ and O_2_^−^) contents in Tuha plants under different altitudes. Results of the H_2_O_2_ content showed that leaves of LA plants contained the lowest H_2_O_2_ levels compared with middle- and high-altitude plants. The H_2_O_2_ content in leaves of LA plants was significantly decreased by 32.28% compared with that in MA plants and by 70.65% compared with that in HA plants (Figure 6A–C). The effect of epidermal hairs on H_2_O_2_ levels of the Tuha plants at different altitudes was further analyzed. In low-, middle- and high-altitude areas, regardless of presence of epidermal hairs, there were no significant differences in the H_2_O_2_ content (Figure 6D).

**Figure 7:**
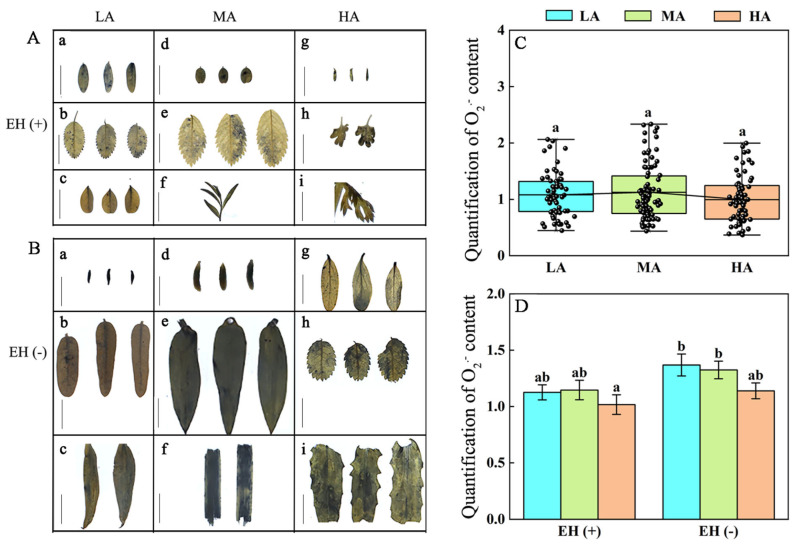
Analysis of superoxide anion (O_2_^−^) in plant leaves at different altitudes in the Tuha Basin. (**A**,**B**) NBT staining of O_2_^−^ content in leaves of plants with (**A**, EH+) or without (**B**, EH−) epidermal hairs at different altitudes. Bar = 1 cm. (**C**) Quantification of O_2_^−^ in plant leaves under different altitudes. (**D**) Quantification of H_2_O_2_ in plants with (EH+) or without (EH−) epidermal hairs at different altitudes. In **A**, **a**: *Oxytropis glabra* from S9 (651 m); **b**: *Rosa multiflora* from S9 (651 m); **c**: *Alhagi camelorum* from S56 (1004 m); **d**: *Sphaerophysa salsula* from S58 (1139 m); **e**: *Rosa laxa* from S58 (1139 m); **f**: *Braya humilis* from S59 (1785 m); **g**: *Descurainia Sophia* from S60 (2093 m); **h**: *Artemisia frigida* from S61 (2459 m); **i**: *Achillea millefolium* from S61 (2459 m). In **B**, **a**: *Lycium ruthenicum* from S7 (461 m); **b**: *Karelinia caspia* from S9 (651 m); **c**: *Takhtajaniantha austriaca* from S9 (651 m); **d**: *Salsola collina* from S54 (1813 m); **e**: *Phragmites australis* from S58 (1139 m); **f**: *Iris tectorum* from S58 (1139 m); **g**: *Polygonum aviculare* from S60 (2093 m); **h**: *Rosa laxa* from S60 (2093 m); **i**: *Thlaspi arvense* from S60 (2093 m). Different letters on the error bar indicate significant difference at *p* < 0.05. LA, low altitude; MA, middle altitude; HA, high altitude.

There were no significant variations in the O_2_^−^ levels in leaves across low-, middle-, and high-altitude regions (Figure 7A–C). Plants with or without epidermal hairs exhibited no significant differences in the O_2_^−^ content under different altitudes (Figure 7D). These findings demonstrate that H_2_O_2_ was significantly induced with increased altitude and UV-B radiation intensity in leaves of plants in the Tuha Basin, whereas O_2_^−^ accumulation showed no significant correlation with the altitude.

### 3.7. Changes in C, N, H, and S Contents in Leaves of Plants from Different Altitudes

**Figure 8:**
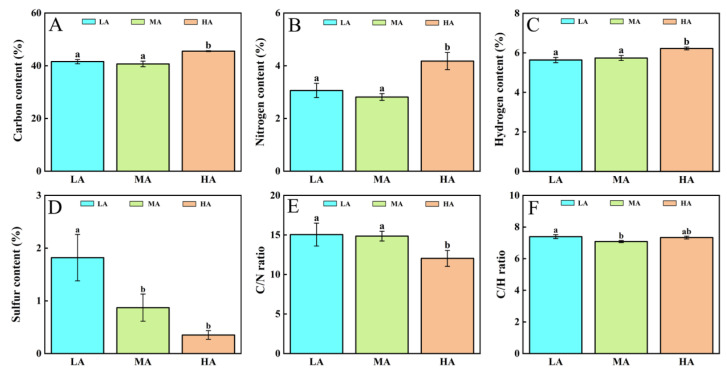
Analysis of organic elements in plant leaves at different altitudes in the Tuha Basin. (**A**) Carbon; (**B**) Nitrogen; (**C**) Hydrogen; (**D**) Sulfur; (**E**) C/N ratio; (**F**) C/H ratio. Different letters on the error bar indicate significant difference at *p* < 0.05. LA, low altitude; MA, middle altitude; HA, high altitude.

The organic element composition in plants reflects resource allocation strategies and responses to environmental changes [52,53,54]. Therefore, we analyzed the contents of four macroelements, i.e., C, N, H, and S in leaves of plants from different altitudes in the Tuha Basin and their variation trends with increased altitudes. The results showed that there were no significant changes in C, N, or H contents from low- to middle-altitude regions. However, from middle- to high-altitude regions, their contents increased significantly by 11.95%, 48.53%, and 8.41%, respectively (Figure 8A–C). In contrast, the S content was the highest in LA plants. Compared with LA plants, the S contents in MA and HA plants were significantly reduced by 50.15% and 80.66%, respectively (Figure 8D).

We further investigated the variation patterns of C/N and C/H ratios in plant leaves from different altitudes. The results showed that the C/N ratio remained stable from low- to middle-altitude regions but decreased significantly by 18.92% under high-altitude regions (Figure 8E). The C/H ratio was significantly reduced by 4.21% from low- to middle-altitude regions; and there was no significant difference from middle- and high-altitude regions (Figure 8F). These results indicate that the contents and the ratios of organic elements in plant leaves undergo significant changes with increased altitudes, which may reflect the responsive mechanism in Tuha pants adaptation to the strong UV-B radiation and high-altitude environments.

## 4. Discussion

As typical sessile organisms, plants have developed complex responsive mechanisms during the long-term evolution to cope with dynamic environmental conditions [55]. Among these, UV-B radiation, one of the key stress factors in the high-altitude regions, can significantly affect plant growth and development by inducing photic damages of DNA, membrane lipid peroxidation, and photosystem inactivation [11,14]. Compared with inland areas in China, the Tuha Basin has longer solar radiation and higher UV-B intensity, especially in mountainous, desert, and Gobi regions [54]. During the long-term evolutionary adaptation, plants in the Tuha Basin have developed a serious of UV-B tolerance mechanisms, forming unique UV-tolerant plant communities. To elucidate the altitude-dependent UV-B adaptation strategies, the UV-B adaptation mechanism of 78 plant species was systematically analyzed through detecting the physiological and morphological indicators under different altitudes in the Tuha Basin, i.e., the levels of UV-B absorbing substances (Fla, Ant), photosynthetic pigments (Chl a, Chl b, Car) and ROS (H_2_O_2_, O_2_^−^), the changes in morphological indicators (epidermal hairs, cuticle, stomata), and organic element (C, N, H, S) contents. We have established a comprehensive evaluation system for UV-B tolerance of plants in the Tuha Basin and evaluated their UV-B tolerance ability. This study aims to systematically reveal the longitudinal adaptation strategies and mechanisms in plant adaptation to changes in altitude and UV-B radiation through investigating and comparing the differences in the microstructure and physiological and biochemical responses of plants at different altitudes in the Tuha Basin.

### 4.1. Plants Adapt to the Altitude-Dependent UV-B Radiation Through Differentially Regulating the Levels of Photo-Protective and UV-B Absorbing Substances in the Tuha Basin

Plant survival under UV-B radiation is achieved through comprehensive mechanisms of exposure limitation, protection, and repair [5]. Phenylpropanoid compounds, serving as “UV-B absorbing sunscreens”, include Fla and polyphenols [20]. They accumulated in the epidermal cells to protect plants from UV-B damage [56,57]. Meanwhile, Ants and Car function as natural antioxidants that play a crucial role in scavenging free radicals and protecting plant tissues from excessive UV-B radiation [19,21,58]. The H_2_O_2_ contents in leaves of various plant species were significantly increased with increased altitude in the Tuha Basin (Figure 6C); whereas the O_2_^−^ content exhibited no significant changes (Figure 7C). These results suggest that high altitudes in the Tuha Basin may cause strong environmental stresses such as enhanced UV-B radiation, low temperature, and hypoxia, which further induce high ROS levels. As a core member of ROS, H_2_O_2_ can cause oxidative damage and disrupt cellular structures [59], while also functions as a signaling molecule involved in plant defensive responses [60]. Thus, the changing H_2_O_2_ level could be one of the essential mechanisms in plant adaptation to high altitude and UV-B radiation in the Tuha Basin.

A total of 78 plant species were investigated in different altitude areas of the Tuha Basin, belonging to 19 families and 42 genera (Tables S1 and S2). The intensity of UV-B radiation is significantly positively correlated with altitudes. Among various plant species in the Tuha Basin, the Fla content was highest in LA plants and lowest in MA plants; comparatively, Ant was highest in HA plants (Figure 1A,B and Table S3). Meanwhile, the contents of Car and Chl (Chl a, Chl b, Chl t) were significantly accumulated with increased altitude in various plant species in the Tuha Basin (Figure 1C–F and Table S3). PCA results further showed that the chlorophyll content (Chl a, Chl b, Chl t), Chl a/Chl b ratio, and the Fla content in low-, middle-, and high-altitude regions of the Tuha Basin cover the majority of information on eight physiological indicators and are important indicators for evaluating UV-B tolerance of plants. Noticeably, with the increased altitude, Chl b, Chl a/Chl b, and Fla play increasingly important roles.

Noticeably, plants in LA regions primarily rely on accumulating Fla and Ant to absorb UV-B radiation (Figure 1A,B and Table S3). The dominant species include *Sophora alopecuroides*, *Phragmites australis*, *Populus euphratica*, *Apocynum venetum*, *Tamarix chinensis*, and *Capparis spinosa* (Table 2). In MA regions, the Fla content in leaves decreased significantly, while Car and Chl contents increased (Figure 1 and Table S3). Car can markedly scavenge free radicals and reduce photo-oxidative damage. The high Chl levels can maintain the photosynthetic performance. Dominant species mainly include *Phragmites australis*, *Pennisetum alopecuroides*, *Carex ovatispioulata*, *Oxytropis glabra*, *Caragana sinica*, *Kali collinum*, *Sophora alopecuroides*, *Lepidium latifolium*, *Krascheninnikovia ceratoides*, *Takhtajaniantha austriaca*, and *Apocynum pictum Schrenk* (Table 2). In HA regions, Fla, Ant, and Chl contents in leaves further increased (Figure 1 and Table S3). Dominant species include *Oxytropis glabra*, *Potentilla chinensis*, *Neotrinia splendens*, *Medicago sativa*, *Agropyron cristatum*, *Chenopodium album*, *Astragalus scaberrimus*, *Iris tectorum*, *Oxytropis aciphylla*, and *Echinops sphaerocephalus*.

Based on these results, we propose that in LA regions, plants mainly accumulate Fla to absorb UV-B radiation, thereby protecting photosynthetic performance and reducing UV-B damage; and Ant may also participate in this process. In MA regions, Car and Chl play an essential role in scavenging UV-B-induced free radicals and reducing photooxidative damage. The elevated chloroplast pigment content facilitates to maintain plant photomorphogenesis and developmental status. In HA regions, further accumulation of Fla, Ant, and chloroplast pigments indicate that plants require high levels of UV-B absorbing compounds/free radical scavengers and photosynthetic pigment levels to maintain their photosynthetic performance and normal development processes. Significantly, the highest content of Fla in LA plants but Chl and Ant in HA plants indicate that the changes in the contents of photo-protective and UV-B absorbing substances induced by altitude-dependent UV-B radiation may be determined by species and comprehensive environmental factors.

### 4.2. The Epidermal Structure and Stomatal Characteristics Coordinately Regulate Plants in Adaptation to the High-Altitude Environments in the Tuha Basin

Epidermal hairs may play important roles in protecting plants from environmental stresses such as heat, low temperature, high UV-B radiation, and insect herbivory [61,62]. The cuticle also plays key roles in drought stress, pathogen resistance, and limiting the cuticle-dependent water loss from leaf surface [63,64]. However, the epidermal hair density in plant leaves of the Tuha Basin decreases with increased altitudes (Figure 2I and Table S4). Moreover, for the same species, the number of epidermal hairs significantly decreases with increased altitudes, for example, *Oxytropis glabra*, *Alhagi sparsifolia*, and *Ephedra sinica* (Figure 2A–H,J). The cuticle thickness also shows a similar trend, and this change is independent of epidermal hair number at the same altitude (Figure 3B,C). Meanwhile, in low-, middle- and high-altitude regions, epidermal hair density does not significantly affect H_2_O_2_ and O_2_^−^ levels in plant leaves (Figure 6 and Figure 7). Due to high consumption of carbon skeletons in the synthesis of wax layers and epidermal hairs, plants in the high-altitude environment of the Tuha Basin tend to prioritize the allocation of limited resources to secondary metabolites rather than structural defense, as evidenced by higher Chl and Ant contents (Figure 1A–F).

UV-B can be perceived by the UV-B photoreceptor UVR8 in plant epidermis, which further triggers UV-B signaling and induces stomatal closure [65]. Thus, the regulation of stomatal characteristics also plays a significant role in plant adaptation to UV-B stress. Our results showed that as altitude increased, the length, width, and area of stomata were significantly increased (Figure 4A,B,D). The enlargement of stomatal aperture helps maintain the diffusion efficiency of O_2_ and CO_2_, to meet the needs of photosynthetic C assimilation and respiratory rate in HA environments. However, the stomatal density exhibited an opposite trend (Figure 4E); and the stomatal length-to-width ratio and SLA were the lowest in MA plants compared with LA and HA plants (Figure 4C). These results indicated that the increase in altitude will induce stomatal enlargement and enhancethe stomatal density and SLA. Plants might reduce the damage induced by a combination of UV-B radiation and high-altitude factors through reducing the stomatal density and SLA to compensate for the changes in epidermal structures in HA environments.

In LA regions, the presence or absence of epidermal hairs had no significant effect on stomatal area, stomatal density, or SLA (Figure 5). However, in MA and HA regions, plants without epidermal hairs had significantly larger areas of stomatal apparatus compared with plants with epidermal hairs (Figure 5B). The decrease in stomatal density or SLA is not related to the epidermal hair number in HA regions (Figure 5C,D). These results indicate that in middle- and high-altitude regions, plants without epidermal hairs tend to develop larger stomata compared to plants with epidermal hairs. The increased stomatal apparatus area, along with decreased density and SLA may contribute to reduced transpiration rate of plants in HA environments.

### 4.3. Plants in the Tuha Basin Reconfigure Element Allocation to Cope with HA Environments

The content of organic elements (C, N) and the C/N ratio in plants can explain the allocation strategy of photosynthetic compounds towards defensive secondary metabolites, i.e., Fla and Ant, under stress conditions. They also reflect the coordinated mechanisms between the antioxidant system and energy metabolism [4,5]. S, an essential macronutrient for plant growth and development, participates in the biosynthesis of cysteine (Cys) and GSH. Cys can be further converted into GSH, a crucial antioxidant involved in ROS scavenging [65]. The results showed that there are no significant changes in C, N, or H contents in plant leaves from low- to middle-altitude regions (Figure 8A–C). However, from middle- to high-altitude regions, their contents increase significantly. In contrast, S content is the highest in LA plants. The C content is closely related to photosynthetic assimilation efficiency [66]. The N content indicates the balance between protein synthesis (growth) and secondary metabolism (defense, [67,68]). These results indicate that plants in the Tuha Basin may synergistically regulate the balance between defense metabolism and growth by optimizing element ratios in their adaptation to different altitudes. Meanwhile, S is also a component of proteins and glucosinolates. In plants grown at high altitudes, allocation of S in different metabolic pathways may shift towards the priority synthesis of specific sulfur-containing compounds, which are crucial for adapting to environmental stressors such as low temperatures, high UV-B radiation, and limited nutrient availability. The decrease in S content may reflect a re-allocation of S resources toward structural components (i.e., proteins). Such re-allocation can enhance the stability of cellular structures, which is crucial for plants to withstand changing environmental conditions across different altitudes in the Tuha Basin.

The balance of C/N metabolism is important for plant growth, development, and the adaptation to the variable environments [69,70]. Our results showed that the C/N ratio remains stable from low- to middle-altitude regions but decreases significantly in HA regions (Figure 8E). A low C/N ratio in plants from HA regions indicates that N might be preferentially allocated to N-containing defensive compounds (such as alkaloids) or key enzymes (i.e., PAL, CHS) to balance growth and stress tolerance [71]. Comparatively, the C/H ratio is significantly reduced from low- to middle-altitude regions; but there is no significant difference between middle- and high-altitudes (Figure 8F). These results indicate that the contents and the ratios of organic elements in plant leaves undergo significant changes with increased altitude, which may reflect the responsive mechanism in the adaptation of Tuha plants to high UV-B radiation and high-altitude conditions. It is well known that a high N metabolism rate is beneficial for reducing photooxidative damage and maintaining optimal photosynthetic performance [43,72]. The significant decrease in C/N ratio in high-altitude areas indicates a shift in plant metabolic patterns. The synergistic regulation of C and N metabolic homeostasis should be an effective strategy for plants to adapt to high-altitude environments in Tuha basin.

## 5. Conclusions

Overall, the contents of Chl t, Chl a, Chl b, and Fla, as well as Chl a/Chl b, are important indicators for evaluating plant tolerance to UV-B at different altitudes of the Tuha Basin. Moreover, with increased altitudes, the roles of Chl b, Chl a/Chl b, and Fla become markedly significant. The epidermal hair density and cuticle thickness in plant leaves decrease with increased altitudes in the Tuha basin; comparatively, H_2_O_2_ is significantly accumulated, but O_2_^−^ content remains unchanged. High altitude significantly increases stomatal apparatus area, density, and SLA. Plants without epidermal hairs have a larger stomatal apparatus area compared with plants with epidermal hairs in both middle- and high-altitude regions. However, the presence or absence of epidermal hairs has no effect on cuticle thickness, H_2_O_2_ and O_2_^−^ levels. The C, N, and H contents are the highest in plant leaves at high altitudes, but the S content and the C/N ratio are the lowest. Taken together, plants can cope with UV-B radiation by synergistically regulating epidermal structures (epidermal hairs, cuticle, and stomatal characteristics), synthesis of secondary metabolites (chloroplast pigments and Fla), and allocation and reconstruction of organic elements (C, N, S) under different altitudes in the Tuha Basin. Further research should separate the altitude-related factors from other environmental variables by controlling UVB intensity to more accurately clarify the mechanisms and strategies of plant adaptation to the increased UV-B radiation with altitude in Tuha Basin.

## Figures and Tables

**Table 1:** Principal component analysis of plant UV-B related traits at different altitudes.

Altitude Type	Principal Components	PC1	PC2	PC3
Low altitude	Fla (n = 71)	0.306	0.227	0.695
	Ant (n = 75)	0.357	0.419	0.581
	Chl a (n = 77)	0.977	0.116	−0.170
	Chl b (n = 77)	0.991	−0.016	−0.116
	Car (n = 75)	0.732	0.610	−0.217
	Chl a/Chl b (n = 73)	0.483	0.714	−0.348
	Chl t/Car (n = 74)	0.696	−0.673	−0.008
	Chl t (n = 77)	0.987	0.050	−0.144
	Characteristic root (λ)	4.385	1.579	1.052
	Contribution rate (%)	54.807	19.732	13.145
	Cumulative rate of contribution (%)	54.807	74.540	87.685
Middle altitude	Fla (n = 84)	−0.486	−0.190	0.744
	Ant (n = 93)	0.539	0.260	0.522
	Chl a (n = 99)	0.975	0.138	0.025
	Chl b (n = 99)	0.985	−0.039	0.030
	Car (n = 99)	0.575	0.772	0.005
	Chl a/Chl b (n = 92)	−0.476	0.828	−0.057
	Chl t/Car (n = 98)	0.539	−0.791	−0.057
	Chl t (n = 99)	0.985	0.049	0.028
	Characteristic root (λ)	4.265	2.034	0.834
	Contribution rate (%)	53.314	25.421	10.430
	Cumulative rate of contribution (%)	53.314	78.735	89.165
High altitude	Fla (n = 63)	0.207	−0.428	0.806
	Ant (n = 98)	0.749	−0.160	0.126
	Chl a (n = 102)	0.877	0.467	−0.014
	Chl b (n = 102)	0.905	−0.110	−0.266
	Car (n = 98)	0.749	−0.338	0.177
	Chl a/Chl b (n = 99)	−0.003	0.929	0.309
	Chl t/Car (n = 95)	−0.042	0.981	0.147
	Chl t (n = 102)	0.946	0.206	−0.142
	Characteristic root (λ)	3.651	2.420	0.906
	Contribution rate (%)	45.635	30.247	11.321
	Cumulative rate of contribution (%)	45.635	75.882	87.203

**Table 2:** Comprehensive evaluation D value and ranking of different species at different altitudes.

Altitude Type	Species	Altitude (m)	D Value	The Resistance to UV
High altitude	*Oxytropis glabra*	2212	0.947	I
	* Potentilla chinensis *	2212	0.933	I
	* Neotrinia splendens *	1907	0.870	II
	* Medicago sativa *	1907	0.859	II
	* Agropyron cristatum *	2212	0.730	II
	* Chenopodium album *	1907	0.670	II
	* Astragalus scaberrimus *	2212	0.597	II
	* Iris tectorum *	2212	0.555	II
	* Oxytropis aciphylla *	2212	0.547	II
	* Echinops sphaerocephalus *	2212	0.501	II
	* Takhtajaniantha austriaca *	2212	0.492	III
	* Halogeton glomeratus *	1907	0.484	III
	* Glaucium squamigerum *	1907	0.460	III
	* Braya humilis *	1907	0.293	IV
Middle altitude	* Phragmites australis *	1144	0.973	I
	* Pennisetum alopecuroides *	1473	0.959	I
	* Carex ovatispioulata *	1473	0.951	I
	* Oxytropis glabra *	1500	0.782	II
	* Caragana sinica *	1500	0.771	II
	* Kali collinum *	1473	0.704	II
	* Sophora alopecuroides *	1144	0.665	II
	* Lepidium latifolium *	1473	0.643	II
	* Krascheninnikovia ceratoides *	1473	0.638	II
	* Takhtajaniantha austriaca *	1473	0.629	II
	* Apocynum pictum *	1144	0.501	II
	* Sympegma regelii *	1473	0.472	III
	* Krascheninnikovia ceratoides *	1500	0.454	III
	* Braya humilis *	1473	0.424	III
	* Ephedra sinica *	1473	0.400	III
	* Halogeton glomeratus *	1473	0.386	III
	* Peganum harmala *	1500	0.364	III
	* Zygophyllum fabago *	1473	0.332	III
	* Zygophyllum xanthoxylum *	1473	0.322	III
	* Neotrinia splendens *	1500	0.279	IV
	* Anabasis brevifolia *	1473	0.253	IV
	* Lycium ruthenicum *	1114	0.224	IV
	* Anabasis aphylla *	1500	0.152	IV
Low altitude	* Sophora alopecuroides *	615	0.913	I
	* Phragmites australis *	461	0.839	II
	* Populus euphratica *	615	0.645	II
	* Apocynum venetum *	510	0.622	II
	* Tamarix chinensis *	510	0.525	II
	* Capparis spinosa *	510	0.503	II
	* Alhagi camelorum *	980	0.467	III
	* Cynanchum acutum subsp *	510	0.368	III
	* Peganum harmala *	980	0.303	III
	* Lycium ruthenicum *	461	0.283	IV
	* Haloxylon ammodendron *	626	0.243	IV
	* Karelinia caspia *	510	0.217	IV

## Data Availability

The original contributions presented in this study are included in the article. Further inquiries can be directed to the corresponding author.

## Supplementary Materials

The following supporting information can be downloaded at: https://www.mdpi.com/article/10.3390/life15091375/s1, Figure S1: Principal component analysis (PCA) of UV-B tolerance related indices in different plant species at different altitudes in the Tuha Basin; Figure S2: Correlation analysis between D value and fuzzy membership function values; Table S1: The information of samples collected in 2023; Table S2: The information of samples collected in 2024; Table S3: Analysis of UV-B tolerance related indices in different plants at different altitudes; Table S4: Analysis of epidermal hair density of plants at different altitudes; Table S5: Analysis of cuticle thickness in plant leaves at different altitudes in the Tuha Basin.

## Abbreviations

Ant	Anthocyanin
C	Carbon
Car	Carotenoid
Chl	Chlorophyll
Fla	Flavonoid
HA	High altitude
H_2_O_2_	Hydrogen oxide
LA	Low altitude
MA	Middle altitude
N	Nitrogen
O_2_^−^	Superoxide anion
PCA	Principal component analysis
ROS	Reactive oxygen species
S	Sulfur
Tuha	Turpan-Hami
UV-B	Ultraviolet B
