# Supplementary material for: Adaptation of Plants to UV-B Radiation with Altitude in Tuha Basin: Synergistic Regulation of Epidermal Structure, Secondary Metabolites, and Organic Element Allocation
Source: Life (Basel). 2025 Aug 29;15(9):1375. doi: 10.3390/life15091375 (PMC12471477; doi:10.3390/life15091375)
Supplement: Supplementary file 1 [file life-15-01375-s001.zip › life-3831624-supplementary.pdf]

Supplemental data

Fig. S1

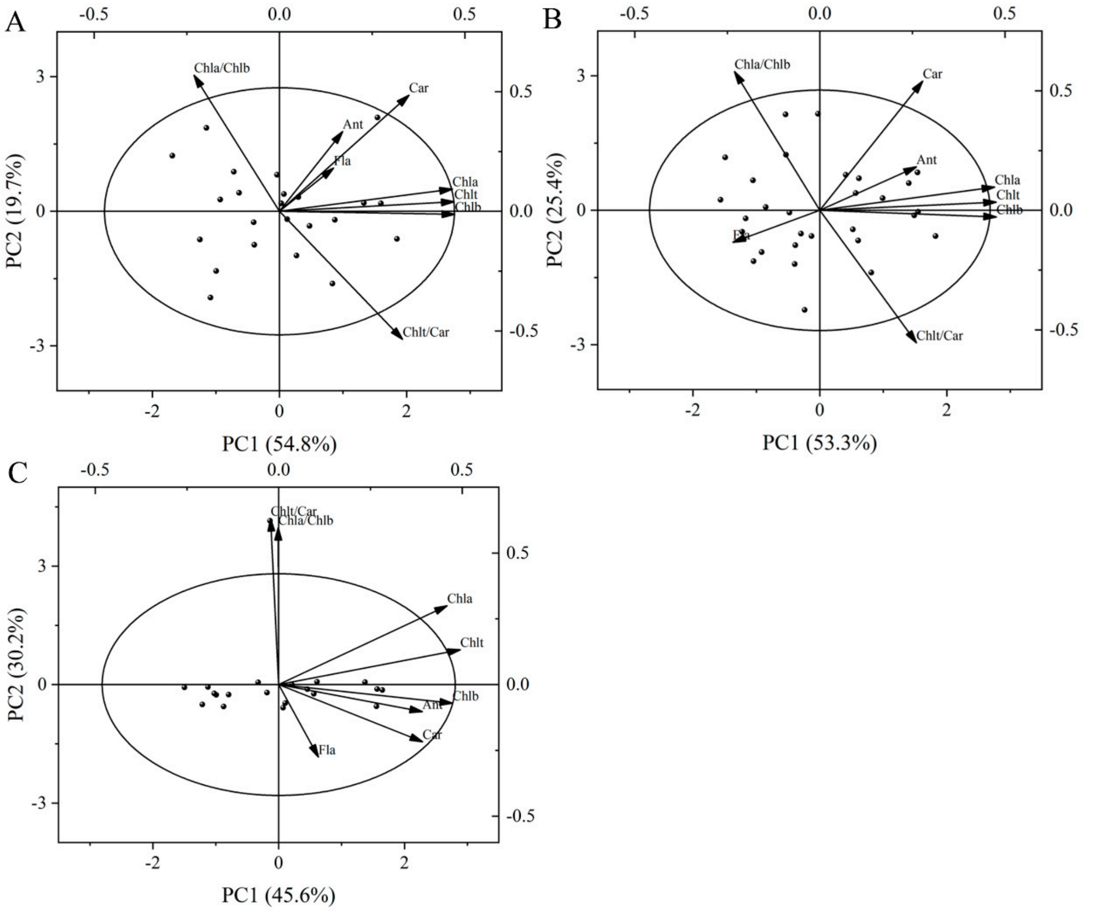

Figure S1. Principal component analysis (PCA) of UV-B tolerance related indexes of various plant species at different altitudes in Tuha Basin. (**A**) Low altitude; (**B**) Middle altitude; (**C**) High altitude.

Fig. S2

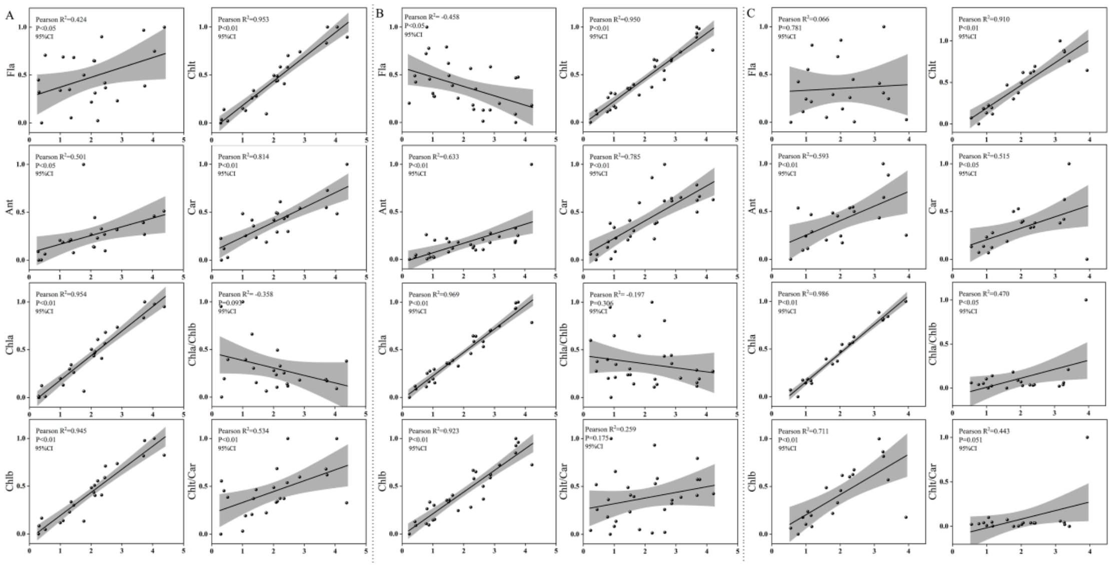

Figure S2. Correlation analysis between D value and fuzzy membership function values. The Regression analysis of fuzzy membership function and D value for the UV-tolerance related indicators at low altitude (**A**), middle altitude (**B**) and high altitude (**C**).

Table S1 The information of samples collected in 2023.

Altitude	Species	Altitude （m）	Longitude	Latitude	Site code
Low-altitude regions	*Ephedra sinica*	461	95°03′52	43°44′45	S7
	Phragmites australis	461	95°03′52	43°44′45	S7
	Populus euphratica	461	95°03′52	43°44′45	S7
	Lycium ruthenicum	461	95°03′52	43°44′45	S7
	Alhagi camelorum	461	95°03′52	43°44′45	S7
	Capparis spinosa	510	90°33′42	43°00′32	S20
	Sophora alopecuroides	510	90°33′42	43°00′32	S20
	Cynanchum sibiricum	510	90°33′42	43°00′32	S20
	Alhagi camelorum	510	90°33′42	43°00′32	S20
	Tamarix chinensis	510	90°33′42	43°00′32	S20
	Populus euphratica	510	90°33′42	43°00′32	S20
	Phragmites australis	510	90°33′42	43°00′32	S20
	Karelinia caspia	510	90°33′42	43°00′32	S20
	Apocynum venetum	510	90°33′42	43°00′32	S20
	Populus euphratica	615	94°07′26	43°58′54	S9
	Lycium ruthenicum	615	94°07′26	43°58′54	S9
	Sophora alopecuroides	615	94°07′26	43°58′54	S9
	Phragmites australis	615	94°07′26	43°58′54	S9
	Alhagi camelorum	626	93°26′11	44°34′54	S12
	Haloxylon ammodendron	626	93°26′11	44°34′54	S12
	Calligonum mongolicum	626	93°26′11	44°34′54	S12
	Iljinia regelii	980	91°35′50	43°42′18	S15
	Peganum harmala	980	91°35′50	43°42′18	S15
	Alhagi camelorum	980	91°35′50	43°42′18	S15

	Apocynum pictum	1144	93°25′59	44°10′05	S11
	Reaumuria songarica	1144	93°25′59	44°10′05	S11
	Sophora alopecuroides	1144	93°25′59	44°10′05	S11
	Lycium ruthenicum	1144	93°25′59	44°10′05	S11
	Phragmites australis	1144	93°25′59	44°10′05	S11
	Zygophyllum xanthoxylum	1473	93°9′46	43°9′43	S2
	Convolvulus tragacanthoides	1473	93°9′46	43°9′43	S2
	Anabasis brevifolia	1473	93°9′46	43°9′43	S2
	Glycyrrhiza uralensis	1473	93°9′46	43°9′43	S2
	Sympegma regelii	1473	93°9′46	43°9′43	S2
	Krascheninnikovia ceratoides	1473	93°9′46	43°9′43	S2
	Lepidium latifolium	1473	93°9′46	43°9′43	S2
	Pennisetum alopecuroides	1473	93°9′46	43°9′43	S2
Middle-altitude regions	*Salicaceae Salix*	1473	93°9′46	43°9′43	S2
	Ephedra sinica	1473	93°9′46	43°9′43	S2
	Carex ovatispioulata	1473	93°9′46	43°9′43	S2
	Zygophyllum fabago	1473	93°9′46	43°9′43	S2
	Takhtajaniantha austriaca	1473	93°9′46	43°9′43	S2
	Halogeton glomeratus	1473	93°9′46	43°9′43	S2
	Braya humilis	1473	93°9′46	43°9′43	S2
	Kali collinum	1473	93°9′46	43°9′43	S2
	Convolvulus gortschakovii	1473	93°9′46	43°9′43	S2
	Caragana sinica	1500	91°35′50	43°42′18	S14
	Anabasis aphylla	1500	91°35′50	43°42′18	S14
	Oxytropis glabra	1500	91°35′50	43°42′18	S14
	Reaumuria songarica	1500	91°35′50	43°42′18	S14
	Peganum harmala	1500	91°35′50	43°42′18	S14

	Neotrinia splendens	1500	91°35′50	43°42′18	S14
	Artemisia L.	1907	93°49′47	43°14′48	S3
	Chenopodium album	1907	93°49′47	43°14′48	S3
	Neotrinia splendens	1907	93°49′47	43°14′48	S3
	Oxytropis glabra	1907	93°49′47	43°14′48	S3
	Medicago sativa	1907	93°49′47	43°14′48	S3
	Rosa laxa	1907	93°49′47	43°14′48	S3
	Glaucium squamigerum	1907	93°49′47	43°14′48	S3
	Halogeton glomeratus	1907	93°49′47	43°14′48	S3
	Braya humilis	1907	93°49′47	43°14′48	S3
	Zygophyllum xanthoxylum	1907	93°49′47	43°14′48	S3
	Kali collinum	1907	93°49′47	43°14′48	S3
	Sympegma regelii	1907	93°49′47	43°14′48	S3
High-altitude regions	*Achnatherum inebrians*	1907	93°49′47	43°14′48	S3
	Corydalis adunca	1907	93°49′47	43°14′48	S3
	Anabasis brevifolia	1907	93°49′47	43°14′48	S3
	Plantago asiatica	1907	93°49′47	43°14′48	S3
	Oxytropis glabra	2212	93°46′39	43°19′10	S4
	Takhtajaniantha austriaca	2212	93°46′39	43°19′10	S4
	Potentilla chinensis	2212	93°46′39	43°19′10	S4
	Agropyron cristatum	2212	93°46′39	43°19′10	S4
	Oxytropis aciphylla	2212	93°46′39	43°19′10	S4
	Astragalus scaberrimus	2212	93°46′39	43°19′10	S4
	Echinops sphaerocephalus	2212	93°46′39	43°19′10	S4
	Iris tectorum	2212	93°46′39	43°19′10	S4
	Taraxacum mongolicum	2212	93°46′39	43°19′10	S4
	Carex ovatispioulata	2212	93°46′39	43°19′10	S4

Table S2 The information of samples collected in 2024.

Altitude	Species	Altitude（m）	Lon.	Lat.	Site
Low-altitude regions	*Zygophyllum fabago*	461	95°03′52	43°44′44	S7
	Alhagi camelorum	461	95°03′52	43°44′44	S7
	Caragana halodendron	461	95°03′52	43°44′44	S7
	Lycium ruthenicum	461	95°03′52	43°44′44	S7
	Rhaponticum repens	461	95°03′52	43°44′44	S7
	Cynanchum sibiricum	461	95°03′52	43°44′44	S7
	Phragmites australis	461	95°03′52	43°44′44	S7
	Ephedra sinica	461	95°03′52	43°44′44	S7
	Tamarix chinensis	651	94°07′26	43°58′54	S9
	Oxytropis glabra	651	94°07′26	43°58′54	S9
	Rosa sp.	651	94°07′26	43°58′54	S9
	Karelinia caspia	651	94°07′26	43°58′54	S9
	Zygophyllum fabago	651	94°07′26	43°58′54	S9
	Takhtajaniantha austriaca	651	94°07′26	43°58′54	S9
	Glycyrrhiza uralensis	651	94°07′26	43°58′54	S9
	Sophora alopecuroides	651	94°07′26	43°58′54	S9
	Iris tectorum	651	94°07′26	43°58′54	S9
	Tamarix chinensis	1004	95°15′10	43°11′34	S56
	Iljinia regelii	1004	95°15′10	43°11′34	S56
	Caragana korshinskii	1004	95°15′10	43°11′34	S56
	Alhagi camelorum	1004	95°15′10	43°11′34	S56
	Lycium ruthenicum	1004	95°15′10	43°11′34	S56
	Reaumuria songarica	1004	95°15′10	43°11′34	S56

	Sympegma regelii	1004	95°15′10	43°11′34	S56
	Phragmites australis	1004	95°15′10	43°11′34	S56
	Reaumuria songarica	1139	93°27′55	44°09′55	S58
	Halogeton glomeratus	1139	93°27′55	44°09′55	S58
	Lycium ruthenicum	1139	93°27′55	44°09′55	S58
	Haloxylon ammodendron	1139	93°27′55	44°09′55	S58
	Iris tectorum	1139	93°27′55	44°09′55	S58
	Sphaerophysa salsula	1139	93°27′55	44°09′55	S58
	Glycyrrhiza uralensis	1139	93°27′55	44°09′55	S58
	Nitraria sphaerocarpa	1139	93°27′55	44°09′55	S58
	Rosa laxa	1139	93°27′55	44°09′55	S58
	Phragmites australis	1139	93°27′55	44°09′55	S58
	Apocynum venetum	1139	93°27′55	44°09′55	S58
	Ephedra sinica	1492	95°33'18	42°16'30	S43
Middle-altitude regions	*Zygophyllum xanthoxylum*	1492	95°33'18	42°16'30	S43
	Allium mongolicum	1785	93°03′41	43°53′51	S59
	Haloxylon ammodendron	1785	93°03′41	43°53′51	S59
	Ephedra sinica	1785	93°03′41	43°53′51	S59
	Caragana sinica	1785	93°03′41	43°53′51	S59
	Peganum harmala	1785	93°03′41	43°53′51	S59
	Braya humilis	1785	93°03′41	43°53′51	S59
	Halogeton glomeratus	1785	93°03′41	43°53′51	S59
	Reaumuria songarica	1785	93°03′41	43°53′51	S59
	Grubovia dasyphylla	1785	93°03′41	43°53′51	S59
	Ephedra sinica	1813	96°50′54	41°49′13	S54
	Caragana leucophloea	1813	96°50′54	41°49′13	S54
	Reaumuria songarica	1813	96°50′54	41°49′13	S54

	Zygophyllum xanthoxylum	1813	96°50′54	41°49′13	S54
	Sympegma regelii	1813	96°50′54	41°49′13	S54
	Kali collinum	1813	96°50′54	41°49′13	S54
	Kalidium foliatum	1813	96°50′54	41°49′13	S54
	Haloxylon ammodendron	1813	96°50′54	41°49′13	S54
	Rosa laxa	2093	93°48′55	43°17′20	S60
	Silene gallica	2093	93°48′55	43°17′20	S60
	Thalictrum aquilegiifolium	2093	93°48′55	43°17′20	S60
	Hedysarum alpinum	2093	93°48′55	43°17′20	S60
	Juniperus pseudosabina	2093	93°48′55	43°17′20	S60
	Thlaspi arvense	2300	93°54'54	43°17'22	S36
	Descurainia sophia	2300	93°54'54	43°17'22	S36
	Polygonum aviculare	2300	93°54'54	43°17'22	S36
	Artemisia dracunculus	2300	93°54'54	43°17'22	S36
	Cirsium arvense	2459	93°56′39	43°17′28	S61
High-altitude regions	*Achillea millefolium*	2459	93°56′39	43°17′28	S61
	Juniperus pseudosabina	2459	93°56′39	43°17′28	S61
	Iris tectorum	2459	93°56′39	43°17′28	S61
	Taraxacum mongolicum	2459	93°56′39	43°17′28	S61
	Argentina anserina	2459	93°56′39	43°17′28	S61
	Equisetum ramosissimum	2459	93°56′39	43°17′28	S61
	Arenaria serpyllifolia	2459	93°56′39	43°17′28	S61
	Thalictrum aquilegiifolium	2459	93°56′39	43°17′28	S61
	Oxytropis glabra	2459	93°56′39	43°17′28	S61
	Artemisia L.	2459	93°56′39	43°17′28	S61
	Artemisia dracunculus	2459	93°56′39	43°17′28	S61

Table S3 Analysis of UV-B tolerance related indexes in the various plants at different altitudes.

Lower altitudes	Intermediate altitude	High altitude

Trait and index	Variation range	Average value	SD	CV	Variation range	Average value	SD	CV	Variation range	Average value	SD	CV
Fla (mg g-1 DW)	5.16-22.95	13.47	5.09	0.38	2.64-21.03	9.56	4.56	0.48	6.79-22.52	11.87	4.16	0.35
Ant (OD_{530} g^{-1} FW)	1.44-8.96	4.24	1.63	0.38	1.32-7.65	3.81	1.57	0.41	0.31-10.71	5.03	2.28	0.45
Chla (μg g^{-1} FW)	22.75-211.81	102.67	50.95	0.50	17.07-265.61	121.18	58.16	0.48	34.92-317.20	143.60	67.73	0.47
Chlb (μg g^{-1} FW)	16.49-201.50	95.39	51.07	0.54	14.72-276.61	112.74	57.95	0.51	33.89-293.11	126.33	60.69	0.48
Car (μg g^{-1} FW)	64.73-378.95	196.77	70.96	0.36	35.09-437.00	226.54	100.96	0.45	12.56-556.60	251.24	134.33	0.53
Chla/Chlb	0.79-1.38	1.09	0.11	0.10	0.87-1.38	1.08	0.10	0.09	0.86-1.64	1.11	0.16	0.15
Chlt (μg g^{-1} FW)	39.23-404.80	198.00	101.65	0.51	31.78-542.05	233.85	115.54	0.49	68.79-577.44	269.84	122.70	0.45
Chlt/Car	0.42-1.61	0.93	0.28	0.30	0.42-2.04	1.06	0.39	0.36	0.43-2.13	1.03	0.39	0.38

Table S4 Analysis of epidermal hair density of plants at different altitudes.

Altitude level	Species	Mean epidermal hair density	Level	Altitude
Low-altitude regions	*Alhagi camelorum*	88.65693826	I	461
	Caragana halodendron	128.1689007	I	461
	Oxytropis glabra	64.70381463	I	651
	Sophora alopecuroides	67.32421986	I	651
	Rosa sp.	114.6365093	I	651
	Caragana korshinskii	24.80852816	II	1004
	Ephedra sinica	13.90443434	III	461
	Glycyrrhiza uralensis	16.61606488	III	651
	Alhagi camelorum	8.291957504	IV	1004
Middle-altitude regions	*Nitraria sphaerocarpa*	72.38726698	I	1139
	Caragana leucophloea	70.6934555	I	1813
	Glycyrrhiza uralensis	20.39766986	II	1139
	Rosa laxa	48.83230468	II	1139
	Grubovia dasyphylla	25.43665214	II	1785
	Caragana sinica	31.26037346	II	1785
	Sphaerophysa salsula	3.062827881	IV	1139
High-altitude regions	*Artemisia L.*	25.77676451	II	2459
	Silene gallica	14.79751533	III	2093
	Descurainia sophia	14.32012638	III	2300
	Achillea millefolium	14.63032043	III	2459
	Hedysarum alpinum	2.157079646	IV	2093
	Argentina anserina	0.857300885	IV	2459
	Oxytropis glabra	2.46941697	IV	2459

Table S5 Analysis of thickness of cuticle in plant leaves at different altitudes in Tuha Basin.

Altitude level	Species	Relative thickness of the waxy layer	Level	Altitude
	Alhagi camelorum	0.76	I	461
	Rhaponticum repens	0.79	I	461
	Cynanchum acutum	1.00	I	461
	Caragana halodendron	0.72	II	461
	Phragmites australis	0.73	II	461
	Takhtajaniantha austriaca	0.59	II	651
	Sophora alopecuroides	0.64	II	651
	Zygophyllum fabago	0.66	II	651
	Iris tectorum	0.66	II	651
	Karelinia caspia	0.70	II	651
	Glycyrrhiza uralensis	0.72	II	651
	Apocynum venetum	0.73	II	651
	Alhagi camelorum	0.58	II	1004
Low altitudes	*Ephedra sinica*	0.29	III	461
	Zygophyllum pterocarpum	0.44	III	461
	Oxytropis glabra	0.26	III	651
	Rosa sp.	0.41	III	651
	Phragmites australis	0.32	III	1004
	Haloxylon ammodendron	0.39	III	1004
	Caragana korshinskii	0.47	III	1004
	Lycium ruthenicum	0.07	IV	461
	Tamarix chinensis	0.09	IV	651
	Iljinia regelii	0.15	IV	1004
	Tamarix chinensis	0.15	IV	1004
	Sympegma regelii	0.17	IV	1004
	Reaumuria songarica	0.17	IV	1004
	Lycium ruthenicum	0.20	IV	1004
	Phragmites australis	0.56	II	1139
	Glycyrrhiza uralensis	0.56	II	1139
	Zygophyllum xanthoxylum	0.59	II	1492
	Ephedra sinica	0.72	II	1492
	Reaumuria songarica	0.28	III	1139
Middle altitude	*Halogeton glomeratus*	0.33	III	1139
	Haloxylon ammodendron	0.40	III	1139
	Sphaerophysa salsula	0.41	III	1139
	Lycium ruthenicum	0.44	III	1139
	Iris tectorum	0.45	III	1139
	Rosa laxa	0.46	III	1139
	Nitraria sphaerocarpa	0.46	III	1139

	Grubovia dasyphylla	0.02	IV	1785
	Caragana sinica	0.03	IV	1785
	Halogeton glomeratus	0.05	IV	1785
	Braya humilis	0.11	IV	1785
	Reaumuria songarica	0.13	IV	1785
	Haloxylon ammodendron	0.15	IV	1785
	Allium mongolicum	0.19	IV	1785
	Ephedra sinica	0.23	IV	1785
	Peganum harmala	0.25	IV	1785
	Sympegma regelii	0.01	IV	1813
	Kali collinum	0.06	IV	1813
	Reaumuria songarica	0.06	IV	1813
	Caragana sinica	0.09	IV	1813
	Kalidium foliatum	0.13	IV	1813
	Zygophyllum xanthoxylum	0.13	IV	1813
	Ephedra sinica	0.19	IV	1813
High altitude	*Rosa laxa*	0.31	III	2093
	Thlaspi arvense	0.37	III	2300
	Equisetum ramosissimum	0.27	III	2459
	Argentina anserina	0.30	III	2459
	Juniperus pseudosabina	0.33	III	2459
	Achillea millefolium	0.36	III	2459
	Cirsium arvense	0.42	III	2459
	Potentilla bifurca	0.42	III	2459
	Taraxacum mongolicum	0.43	III	2459
	Mulgedium tataricum	0.45	III	2459
	Galxima maritima	0.49	III	2459
	Iris tectorum	0.50	III	2459
	Hedysarum alpinum	0.09	IV	2093
	Silene gallica	0.17	IV	2093
	Artemisia annua	0.17	IV	2093
	Juniperus pseudosabina	0.17	IV	2093
	Thalictrum aquilegiifolium	0.23	IV	2093
	Descurainia sophia	0.00	IV	2300
	Polygonum aviculare	0.18	IV	2300
	Artemisia dracunculus	0.21	IV	2300
	Oxytropis glabra	0.11	IV	2459
	Arenaria serpyllifolia	0.18	IV	2459
	Artemisia L.	0.19	IV	2459
	Thalictrum aquilegiifolium	0.24	IV	2459
